# *MDNA*: a software module for DNA structure generation and analysis

**DOI:** 10.1093/nar/gkag549

**Published:** 2026-06-02

**Authors:** Thor van Heesch, Enrico Skoruppa, Peter G Bolhuis, Helmut Schiessel, Jocelyne Vreede

**Affiliations:** Van’t Hoff Institute for Molecular Sciences, University of Amsterdam, Science Park 904, 1098 XH Amsterdam, Netherlands; Cluster of Excellence Physics of Life, TU Dresden, Helmholtzstr. 10, 01069 Dresden, Germany; Van’t Hoff Institute for Molecular Sciences, University of Amsterdam, Science Park 904, 1098 XH Amsterdam, Netherlands; Cluster of Excellence Physics of Life, TU Dresden, Helmholtzstr. 10, 01069 Dresden, Germany; Van’t Hoff Institute for Molecular Sciences, University of Amsterdam, Science Park 904, 1098 XH Amsterdam, Netherlands

## Abstract

Exploring the dynamical and structural properties of molecular complexes involving DNA is a fundamentally important aspect of understanding many biological processes. Although tools exist for modeling linear DNA and simple complexes, significant challenges remain in generating intricate biomolecular assemblies and incorporating biologically relevant modifications. These limitations restrict the ability to create accurate starting configurations for advanced molecular simulation studies. Here, we introduce MDNA, a molecular modeling toolkit that bridges these gaps by enabling the construction and analysis of complex DNA structures. MDNA provides a versatile solution to generate DNA shapes using a spline-based mapping technique that enables the construction of DNA configurations with arbitrary shapes. Key features include support for (non-)canonical base modifications, such as Watson–Crick–Franklin to Hoogsteen transitions, DNA methylation, and the ability to refine structures using Monte Carlo minimization. The toolkit also provides geometric analysis tools based on rigid body formalism to evaluate DNA structures and trajectories. Together, these features enable users to model and analyze DNA configurations in high detail with a modular Python interface. By integrating structure generation and analysis into a single workflow, MDNA facilitates the study of DNA–protein interactions, supporting new insights into DNA dynamics and molecular simulations.

## Graphical Abstract

**Graphical Abstract:**
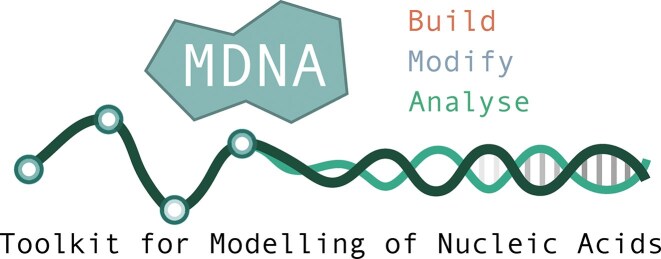

## Introduction

Techniques such as X-ray crystallography, NMR, and cryo-EM are indispensable for obtaining high-resolution structures of biomolecules; however, they face limitations when applied to larger complexes or highly dynamic systems, where obtaining complete atomic detail and capturing transient states can be challenging. Predictive tools such as AlphaFold and RoseTTAFold have expanded our ability to model protein structures from sequence data [1–3], but reliable predictions are generally restricted to single proteins or smaller complexes, as accuracy depends heavily on the availability of high-quality input data, a significant limitation for large, heterogeneous assemblies. Molecular dynamics (MD) simulations provide a complementary approach, capturing the structure and dynamic behavior of biomolecules on biologically relevant timescales [4,5]. The setup of MD simulations requires tools that can generate molecular configurations and topologies in a consistent and flexible manner. Although structures for single proteins or small DNA–protein complexes can be derived experimentally or computationally, constructing larger molecular models containing DNA remains a significant challenge. Models of linear double-stranded DNA (dsDNA) of any sequence can be easily generated, but these approaches are inadequate for more intricate systems, such as protein–DNA complexes, DNA loops, or non-linear DNA topologies. Existing tools often lack the flexibility to construct such structures or to incorporate biologically relevant features like non-canonical bases, methylation patterns, or specific base pairing configurations.

Programs like Avogadro [6] and UCSF ChimeraX [7] allow users to build linear nucleic acid sequences. The user can set the number of base pairs per helical turn or instruct the software to generate the A- or B-form of dsDNA. PyMOL [8] extends these functionalities by enabling chemical customization of DNA or RNA structures and integrating nucleic acids into existing molecular assemblies. The module DSSR (Dissecting the Spatial Structure of RNA) [9] is integrated into PyMOL, extending its capabilities for molecular visualization and structural analysis [10,11]. The proto-Nucleic Acid Builder (pNAB) supports the construction and geometry optimization of diverse nucleic acid analogs with alternative backbones and nucleobases [12]. It is well-suited for generating initial models for MD simulations, particularly for small structures or novel backbone chemistries, though with limited spatial control. Although programs like Avogadro, UCSF ChimeraX, and PyMOL are well-suited for basic nucleic acid structure modeling, including base pair mutations and sequence-to-structure modules to generate linear DNA, they may be less adaptable for constructing more intricate DNA configurations with specific shapes or topological properties.

Building on the foundation of rigid body formalism to describe DNA conformations [13], another class of tools extends these principles to the construction and modeling of larger free-form DNA configurations. By treating each DNA base as a rigid entity with defined spatial positioning and orientation, these tools use geometric parameters to not only describe but also construct DNA structures. For example, the 3DNA software [14,15] allows users to precisely reconstruct molecular structures using the complete set of rigid base parameters as input but lacks the features to specify shapes directly through these descriptors. Using mathematical descriptions of helical parameters, the VMD-based tool VDNA [16] supports the creation of a range of structures from linear to circular DNA and even complex models such as nucleosome superhelices. However, the functionality of VDNA is confined primarily to a graphical user interface within VMD [17], which can hinder reproducibility in large-scale studies. Similarly, web-based tools such as 3Dart and CGeNArateWeb enable users to build 3D structural models of DNA [18,19]. Alternatively, emDNA [20] is designed to model and optimize DNA loops and minicircles at the base pair level, integrating experimental configurations and considering sequence-dependent features to minimize elastic energy. Although emDNA provides tools to minimize energy and build DNA models at base pair resolution, it depends on 3DNA or DSSR to map optimized structures in atomic detail [9,11,14], which can complicate integration into broader simulation workflows.

On the other hand, coarse-grained models like oxDNA [21–25] and tools like Polyply [26] and mrdna [27] are designed to simulate DNA nanostructures and offer powerful capabilities for modeling large-scale systems, such as MD simulations of the entire minimal cell [26,28]. In addition, OxView [29], a tool for visualization and analysis of DNA nanostructures, provides an intuitive free-form editing tool to prepare models for simulation in the oxDNA engine, which users can export to other formats using tacoxDNA [30]. These tools, however, do not offer a solid analysis toolbox for detailed structural analysis of DNA conformations at all-atom resolution.

Instead, 3DNA [14,15] and Curves+ [31] have been the foundation for making the rigid body formalism accessible and consistent for analyzing nucleic acid structures and have significantly advanced understanding of DNA dynamics and flexibility [13,32]. Subsequently, tools such as do_x3dna [33] and NAFlex [34] extend the capabilities of 3DNA and Curves+ by facilitating the analysis of MD trajectories and enabling calculations of DNA’s global helical axis, bending fluctuations, and elastic properties. Biobb_dna, part of the BioExcel building blocks ecosystem [35], aims to improve accessibility by providing parsing and processing for output files from tools such as Canal or Curves+ [31,36,37]. SerraNA [38,39] utilizes a Length-Dependent Elastic Model to analyze simulation data, focusing on DNA bendability and linking molecular fluctuations to macroscopic elastic behavior. Although all of these tools excel at analyzing nucleic acid structures and offer valuable insights into DNA dynamics, they are not designed primarily to generate large, heterogeneous assemblies containing DNA. In terms of analysis of DNA structure within MD simulations, libraries such as MDAnalysis [40], PyTraj [41], and MDTraj [42] provide general-purpose tools for trajectory analysis. Although they offer flexibility and efficiency in handling simulation data, they lack specialized functions for nucleic acid rigid body analysis. Although MDAnalysis previously contained a wrapper for 3DNA, this module is no longer maintained due to licensing restrictions [40]. Other libraries like Bio3D [43], HTMD [44], and VIAMD [45] offer interactive and batch analysis capabilities but do not specifically address the needs of nucleic acid simulations.

To address these challenges, we present MDNA: a software module for DNA structure generation and analysis. MDNA is a Python-based toolkit that integrates DNA structure generation and analysis into a unified framework suitable for molecular dynamics workflows. Our tool employs a spline-based mapping technique to generate accurate DNA structures using Monte Carlo (MC) minimization, supporting features such as the incorporation of non-canonical bases, manipulation of base pairing configurations (e.g. Watson–Crick–Franklin to Hoogsteen), addition of methylation patterns, and the extension or merging of existing DNA models. Furthermore, MDNA integrates post-simulation analysis tools based on a well-established rigid body formalism [13], facilitating direct computation of DNA geometric properties within the MD simulation framework.

By integrating structure generation and analysis tools into a single workflow, MDNA simplifies the process of preparing and analyzing DNA structures for molecular dynamics simulations. This integration facilitates the modeling of complex DNA systems that were previously challenging to construct and analyze, potentially broadening the scope of MD simulations in nucleic acid research. In the following sections, we detail the functionalities, applications, and advantages of the MDNA toolkit.

## Materials and methods

### Overview, usage, and initialization of MDNA

**Figure 1.**
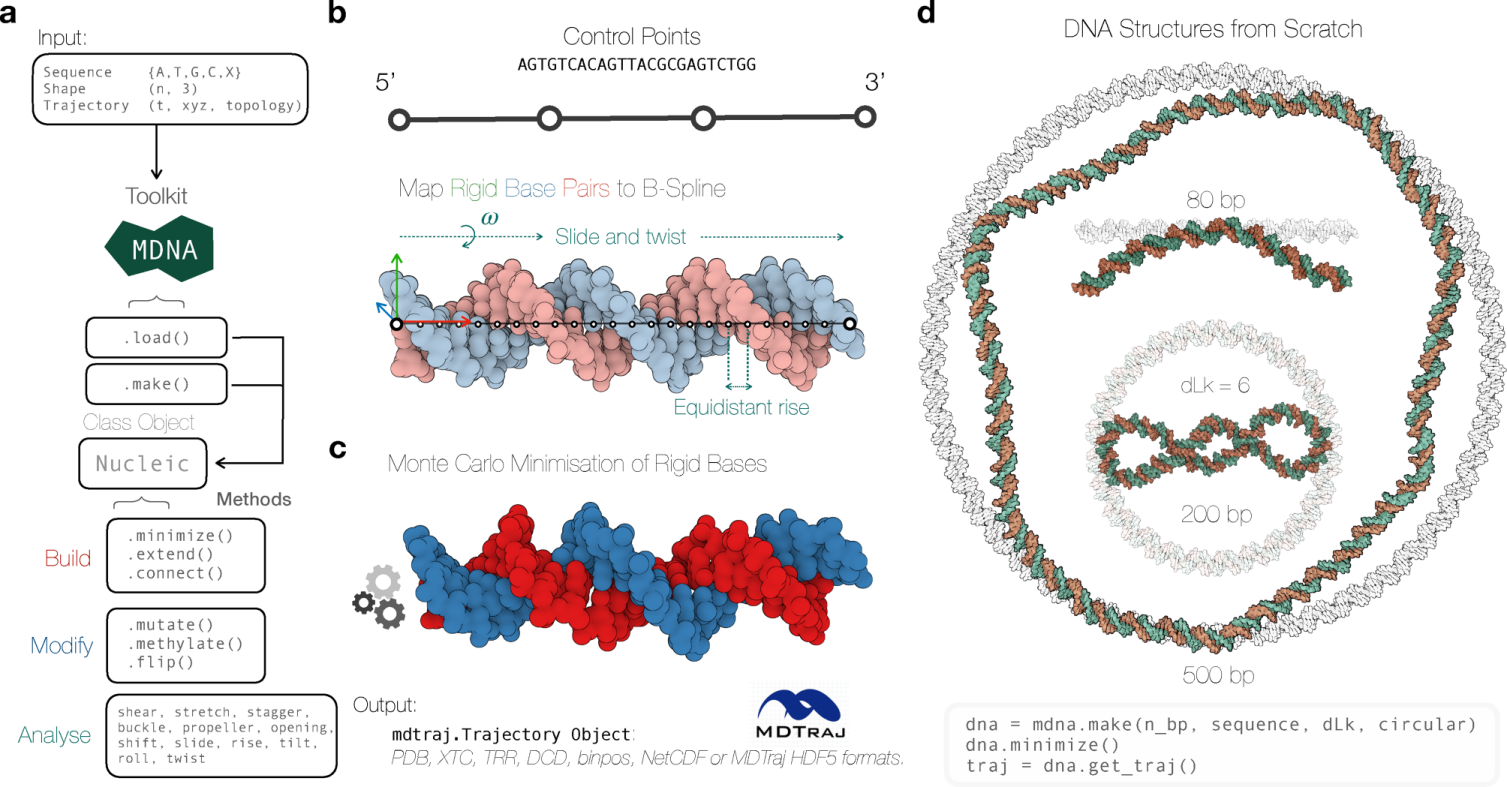
**(A**) A workflow diagram depicting the capabilities of the MDNA toolkit. The diagram begins with input options—sequence (e.g. {A, T, G, C, X}, with X referring to non-canonical base codes), shape, and trajectory—and proceeds to show the toolkit’s central class, Nucleic, which incorporates the functions load() and make() to initialize a Nucleic object. The diagram outlines how this object can be manipulated through categorized methods: *Build* (e.g. minimize(), extend(), connect()), *Modify* (e.g. mutate(), methylate(), flip()), and *Analyze* (e.g. measuring rigid base pair parameters). (**B**) An illustration of mapping a sequence to a B-spline using control points, demonstrating how rigid base pairs are aligned to the spline to create an initial DNA structure. (**C**) Visualization of the minimized DNA structure resulting from Monte Carlo minimization of rigid base steps, with output formats available via MDTraj-compatible formats (e.g. *pdb, xtc, trr*). (**D**) Three examples of pre-minimized spline-based structures (transparent) compared with minimized structures (shown in orange and green): a 500 bp minicircle, an 80 bp linear strand, and a 200 bp minicircle with a dLk = 6, emphasizing writhe conservation. Molecular representations are visualized using Mol* Viewer [46].

The MDNA toolkit streamlines the construction of structural models of dsDNA and the analysis of dsDNA structures and MD trajectories using the rigid base formalism [13]. MDNA serves a broad purpose, facilitating tasks from the calculation of rigid base parameters to the generation, editing, and building of complex DNA structures; see Fig. 1A for a schematic data diagram containing an overview of methods. MDNA emphasizes flexibility, efficiency, and interoperability. To achieve these goals, MDNA leverages MDTraj to bridge MD data with the statistical analysis and scientific visualization ecosystem in Python [42]. MDTraj supports a wide range of MD data formats and calculations, providing a well-tested framework upon which MDNA builds. At the heart of MDNA is the Nucleic class, which unifies both the generation and editing of DNA structures and trajectory analysis. The Nucleic class contains the properties of the DNA structure along with its reference frames and trajectory information, providing methods for further manipulation and analysis.

Initialization of the Nucleic class is achieved through the mdna.make() method, which generates a new DNA structure, or the mdna.load() method, which loads existing DNA structures or trajectories. Once initialized, the Nucleic class offers various methods such as .minimize(), .mutate(), .methylate(), .flip(), .extend(), .connect(). These methods allow users to manipulate DNA structures, introduce mutations, or join DNA fragments. The class also enables the retrieval of topological connectivity and rigid base parameters for structural analysis. For integration with other simulation tools, the .get_traj() method retrieves an MDTraj object of the current DNA configuration. This output can be used directly to start MD simulations in OpenMM [47], or can be exported to various formats, including *pdb, xtc, trr, dcd, binpos, netcdf, mdcrd, prmtop*, and more. In the following, we demonstrate how MDNA facilitates seamless integration of these functions into a cohesive workflow. In the Supplementary Information, we have included a glossary of terms in Supplementary Table S1 as well as a visual overview in Supplementary Fig. S2.

The mdna.load() function is the initial step for loading DNA structures and trajectories using MDTraj [42] or base step reference frames along with a nucleotide sequence. For trajectory inputs, the function extracts the DNA sequence, determines the number of base pairs, and if the MDTraj trajectory contains multiple DNA strands, allows the user to specify the chain IDs of the strand of interest. Additionally, unless explicitly specified otherwise, a DNA structure is classified as circular when the distance between the origins of the first and last base pair is below 1 nm and the sequence contains >20 base pairs. The sequence-length requirement serves as a conservative safeguard to prevent short fragments with coincidentally close ends from being misclassified as circular. For reference frame inputs, the function verifies the dimensionality and consistency of the data with the provided sequence, checking the number of time frames (t), the number of base pairs (n_bp), and the origin together with the orthogonal triad (t, n_bp, 4, 3). This initialization process ensures a consistent DNA representation and returns a Nucleic object.

### Structure generation and minimization

DNA structure generation in MDNA is guided by a flexible spline-mapping protocol (see Supplementary Sections S1 and S2) that enables the creation of atomic-resolution models of varying complexity. Using geometric input such as control points, sequences, and topological properties, MDNA provides users with precise control over DNA shapes, ranging from linear strands to complex loops. Energy minimization is integrated as an optional feature, ensuring biologically relevant configurations through Monte Carlo optimization (see Supplementary Section S3 and Supplementary Fig. S1). This capability opens avenues for modeling unique and non-trivial DNA structures for molecular dynamics simulations.

The core function to initialize the Nucleic class is mdna.make(), which can generate DNA structures from scratch. This method allows flexibility and precision in the production of DNA models by accepting optional input arguments for nucleotide sequence, the number of base pairs (n_bp), topology (circular), adjustment in linking number (dLk), and shape definition (control_points). The control_points are sets of *xyz* coordinates used to determine the shape of a spline curve by specifying a series of interpolation points through which the curve passes. Input can be a custom set of points from a parametric function that describes the desired shape (e.g., an ellipse, superhelix, trefoil knot, etc.), with the only requirement that at least four control points for the interpolation of the spline curve be provided. Once the spline is fitted through the control points, mdna.make() equidistantly distributes the positions of the base pairs along the spline using a spacing of 0.34 nm, which corresponds to the average rise between base pairs. The base pair positions and the tangents of the spline’s space curve are used to construct the base pair reference frames (see Supplementary Section S1). If a target number of base pairs n_bp is specified, the spacing is rescaled along the fixed spline geometry; otherwise, it is set by the spline arc length, with any small deviations smoothly distributed and reported to the user. Starting from the 5′ end, the subsequent base pair reference frames are twisted with respect to the previous base pair to ensure each turn of the double helix contains 10.5 base pairs, resulting in the Darboux frame representation of the DNA structure (see Supplementary Section S2). The user can modify the number of turns per base pair. Circular DNA has a closed topology and may have excess or reduced twist, which is distributed over all base pairs in the sequence. Using the input sequence, the atomic reference coordinates are assigned to the Darboux frame, generating the topological structural information to initialize an MDTraj object (see Supplementary Section S2). All input arguments are optional; if only a DNA sequence is provided, the default settings generate a linear DNA geometry. Users can also control the number of base pairs to scale the shape and adjust the twist via the linking number difference (dLk). The writhe $\mathrm{Wr}$ of the spline backbone is computed at construction time. The supplied linking number difference $\mathtt {dLk}$ (i.e. the change in $\mathrm{Lk} = \mathrm{Tw} + \mathrm{Wr}$ relative to the relaxed state) is then imposed by adjusting the initial twist $\mathrm{Tw}_0$ as $\mathrm{Tw}_\mathrm{new} = \mathrm{Tw}_0 + \mathtt {dLk} - \mathrm{Wr}$, consistent with the White–Fuller theorem. This ensures that the requested linking number difference is correctly realized regardless of any writhe present in the initial shape, see Supplementary Section S5 for more details on linking numbers in DNA. This functionality ensures that the generated DNA structures can be tailored to specific experimental or theoretical requirements. If a closed topology is desired without a specified shape, a circular configuration is generated.

The Nucleic instance from mdna.make() contains by default B-DNA geometries without thermal fluctuations as specified by the control points. To optimize and minimize the energy of the given structure, the nucleic instance contains the .minimize() function, which is designed to relax the DNA structure using MC simulations (see Supplementary Section S3 for implementation details). This relaxation process targets a low-energy configuration by minimizing elastic deformations and steric clashes, modeled through an elastic Hamiltonian and hard sphere potentials. Users can tune the minimization process by adjusting the temperature, excluded volume parameters, and fixing specific base orientations. For example, by keeping the position and orientation of the ends of the DNA strand fixed or by providing a list of base step indices whose orientation and position remain fixed during minimization. When importing a trajectory, a selected snapshot (or time point) can be minimized using the MC model. By default, convergence is assessed by fitting the total elastic energy decay (or writhe) to an exponential function, and the simulation is considered equilibrated once the system has relaxed over a characteristic decay scale, helping to avoid trapping in local minima. If topological properties such as the linking number need to be conserved, the energy is minimized. Equilibrating the writhe relies on checking whether the energy starts to fluctuate around a fixed value, as this process involves a barrier crossing. Figure 1B shows a visual illustration of the spline mapping to construct a DNA structure, as well as the second (optional) stage of MC energy minimization (Fig. 1C). Finally, Fig. 1D presents example DNA structures generated using mdna.make() and subsequently minimized with the MC module. The examples include an 80 bp linear strand, a 200 bp minicircle with dLk set to 6, and a 500 bp minicircle. In the minimized structure of the 200 bp minicircle, we clearly observe the crossings (writhe) in the DNA, which is the result of the conservation of the change in linking number during the MC minimization. Importantly, by constraining the ends of the DNA and or specifying a linking number difference (dLk), MDNA can generate and relax plectonemic configurations, making it possible to model supercoiled DNA geometries prior to more extensive molecular dynamics or Monte Carlo simulations. The computational cost of structure generation and minimization up to 1000 base pairs is negligible and can be done within a minute on an everyday notebook.

### Sequence library and nucleotide modifications

MDNA provides an extensive sequence library that supports the modeling of canonical and non-canonical nucleobases, enabling the exploration of diverse structural properties of DNA. This feature allows researchers to introduce mutations, methylation patterns, and novel base analogs into their models, facilitating studies on topics ranging from genetic mutations to synthetic biology. The toolkit’s library extends to fluorescent and hydrophobic bases, as well as orthogonal systems like Hachimoji DNA. The .mutate() module changes nucleobases in a DNA structure, supporting both complementary and non-complementary pairings. The module includes the canonical bases adenine (A), thymine (T), guanine (G), cytosine (C), and uracil (U) as well as various noncanonical base analogs. The Hachimoji DNA system is available, consisting of four extra synthetic nucleotides forming orthogonal pairs: B-S and P-Z (PDB: 6MIG, 6MIK, and 6MIH) [49]. The library also includes the hydrophobic base pair d5SICS–dNaM [50], an example of unnatural base pairs that interact without hydrogen bonds (PDB: 3SV3 and 4C8M) [51,52]. Furthermore, two fluorescent bases: 2-aminopurine [53] and the tricyclic cytosine analog are available in the nucleotide sequence library [54], both noted for their fluorescent properties in different environments essential in fluorescence studies in nucleic acid research [55] (PDB: 2KV0 and 1TUQ) [56,57]. Note that parameters for all xenogenic nucleobases are not yet available in MD force fields, as this is an ongoing community effort. However, general force fields, such as GAFF(2) and CGenFF [58,59], are available and can often be sufficient. Alternatively, parameters for individual nucleobases can be derived as needed; for example, 2-aminopurine (2AP) parameters based on quantum chemical DFT calculations are available [60], and tools such as modXNA allow for the derivation and construction of modified nucleotides compatible with Amber force fields [61]. See Table 1 for an overview of all available nucleobases in MDNA.

**Table 1.** List of available nucleobases with type, name, code, and complementary base pairing matches

Name	Code	Complementary	Type
Adenosine	A	T	Canoncial
Thymine	T	A	Canonical
Guanine	G	C	Canonical
Cytosine	C	G	Canonical
Uracil	U	A	Canonical
2-Aminopurine	E	T	Fluorescent
Tricyclic cytosine	D	G	Fluorescent
A-analogue	B	T,D	Hachimoji
T-analogue	D	A,B	Hachimoji
G-analogue	Z	C,P	Hachimoji
C-analogue	P	G,Z	Hachimoji
d5SICS	L	M	Hydrophobic
dNaM	M	L	Hydrophobic

**Figure 2.**
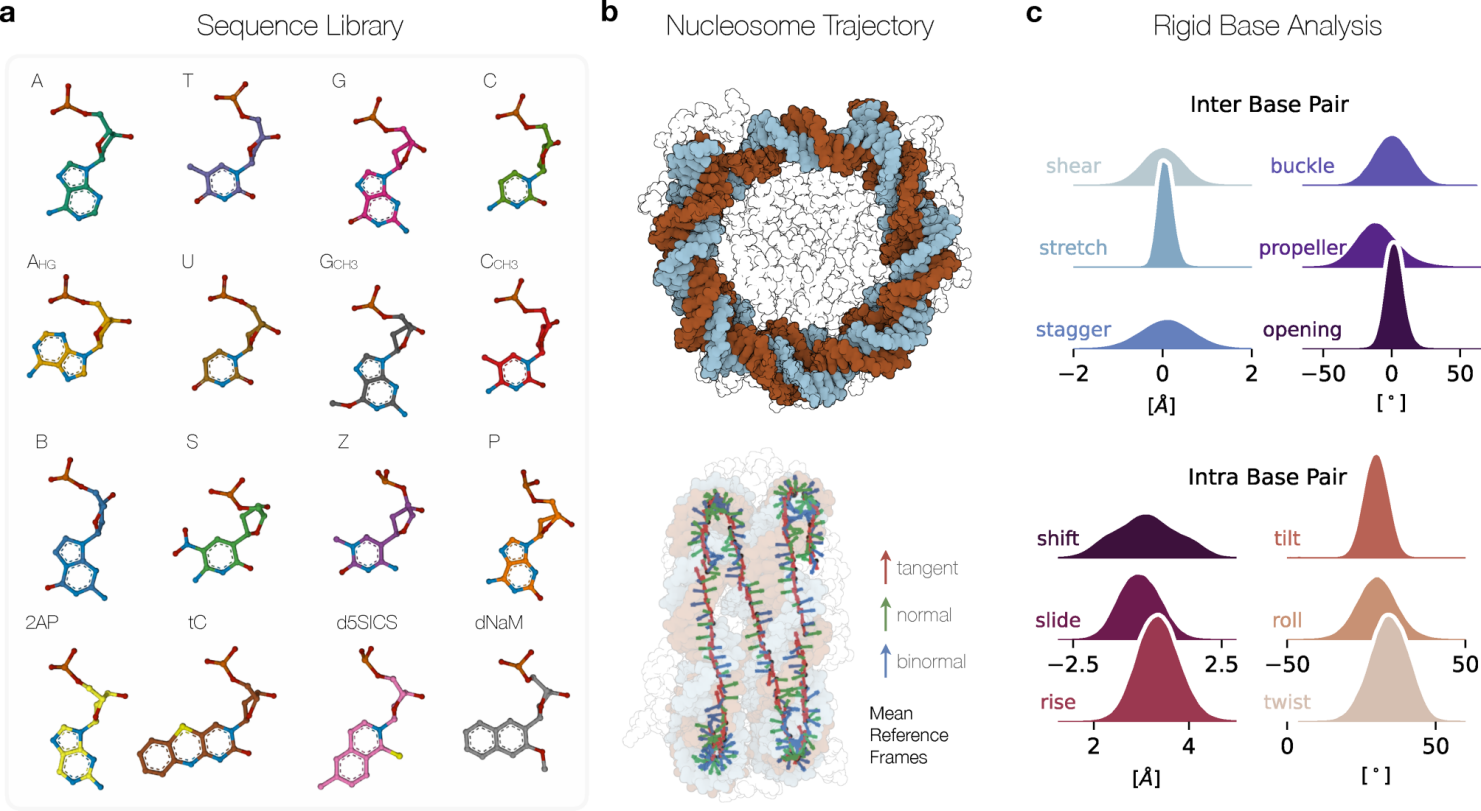
**A**) Sequence library showing molecular representations of the available nucleobases mutations and modifications in the MDNA toolkit. Each nucleobase is annotated with its letter code or acronym. The first row shows the four canonical dsDNA bases, the second row Adenosine in Hoogsteen conformation, followed by Uracil, methylated Guanine, and Cytosine. The third row shows the Hachimoji base analogs: B, S, Z, and P respectively. The last row shows two fluorescent nucleobases 2-amino purine (2AP) and tri cytocine (tC), and the hydrophobic nucleobase set d5SICS and dNaM. **(B**) Front view of a nucleosome core particle with 147 bp DNA and side view of the Darboux frame of the DNA wrapped around the histone showing the mean reference frame of each basepair (PDB: 1KX5 [48]). (**C**) Cumulative rigid base parameter distributions computed with *MDNA* of the nucleosome trajectory of 100 ns. Molecular representations are visualized with Mol* Viewer [46]. (alt text) Library of available nucleobases in MDNA.

The .flip() command rotates the nucleobases around the glycosidic bond that connects the nucleobase to the DNA sugar backbone. By default, nucleobases rotate 180 degrees, shifting from a Watson–Crick–Franklin (WCF) to a Hoogsteen (HG) configuration. The HG base pairing motif, an increasingly recognized structural property of DNA structures, involves a rotation of purine from an anti-to-syn orientation [62]. In this geometry, 5-ring purine, rather than the 6-ring, forms a hydrogen bond with the pyrimidine. HG configurations are more common in AT base pairs, with GC pairs requiring protonated cytosine to accommodate this bonding pattern [63]. The function .methylate() allows users to methylate C or G bases, with the option to automatically methylate cytosines at the CpG sites, where most DNA methylation occurs [64]. Figure 2A shows the extensive sequence library in MDNA, featuring the canonical dsDNA bases, nucleotide modifications and artificial bases. The molecular representations visualized highlight the toolkit’s ability to handle a broad range of DNA structural variations, aiding researchers in exploring the effects of these structural modifications. Both the .flip() and .methylate() only require a list of nucleobase indices as input to indicate which base to flip or methylate.

### Rigid base analysis modules

We offer the first native Python implementation to calculate the rigid base parameters of the structural structure. For intra-base pair movements, they detail rotations (buckle, propeller, and opening) and translations (shear, stretch, and stagger), capturing the nuances of individual base pair positioning. For inter-base pair steps, they define rotations (tilt, roll, and twist) and translations (shift, slide, and rise), providing the spatial and rotational relationships between adjacent base pairs, also known as step parameters. Previously, this required software installations compiling with x3DNA, Curves+/Canal or the dependency on web services for MD trajectory analysis [33,37]. Users can extract these parameters by providing an MDTraj object in the mdna.compute_rigid_parameters() function or use Nucleic class *getters* to obtain a Numpy array (shape: t, n_bp, 12) representing the relative translation and rotation between each base pair, with t the number of trajectory time frames, and n_bp the number of base pairs. The Python implementation uses vectorized arrays using numpy [71]. We used the theoretical framework described in Curves+ [31] and Petkevičiūtė *et al*. to calculate the rigid base parameters [72]. See Supplementary Section S4 for the calculation of rigid parameters. Figure 2B and C illustrates the analysis of rigid base parameters of an MD trajectory of 250 ns of the Nucleosome Core Particle (PDB: 1KX5). Rigid base parameters enable the calculation of global DNA properties, such as the persistence length, which indicates the stiffness of the DNA derived from correlations in base pair orientations; higher correlations suggest a greater stiffness of the DNA strand [73,74]. Furthermore, global tilt and roll parameters help to compute the total curvature of the DNA helix, providing information on the flexibility of the DNA [75]. In addition to the rigid base parameters, MDNA includes the mdna.compute_linking_number() method to calculate the linking number, an important topological property of DNA (see Supplementary Section S5). All of these properties are essential structural metrics for understanding the supercoiling, bending, and interactions of DNA with proteins.

## Results and discussion

### Building biomolecular assemblies

**Figure 3.**
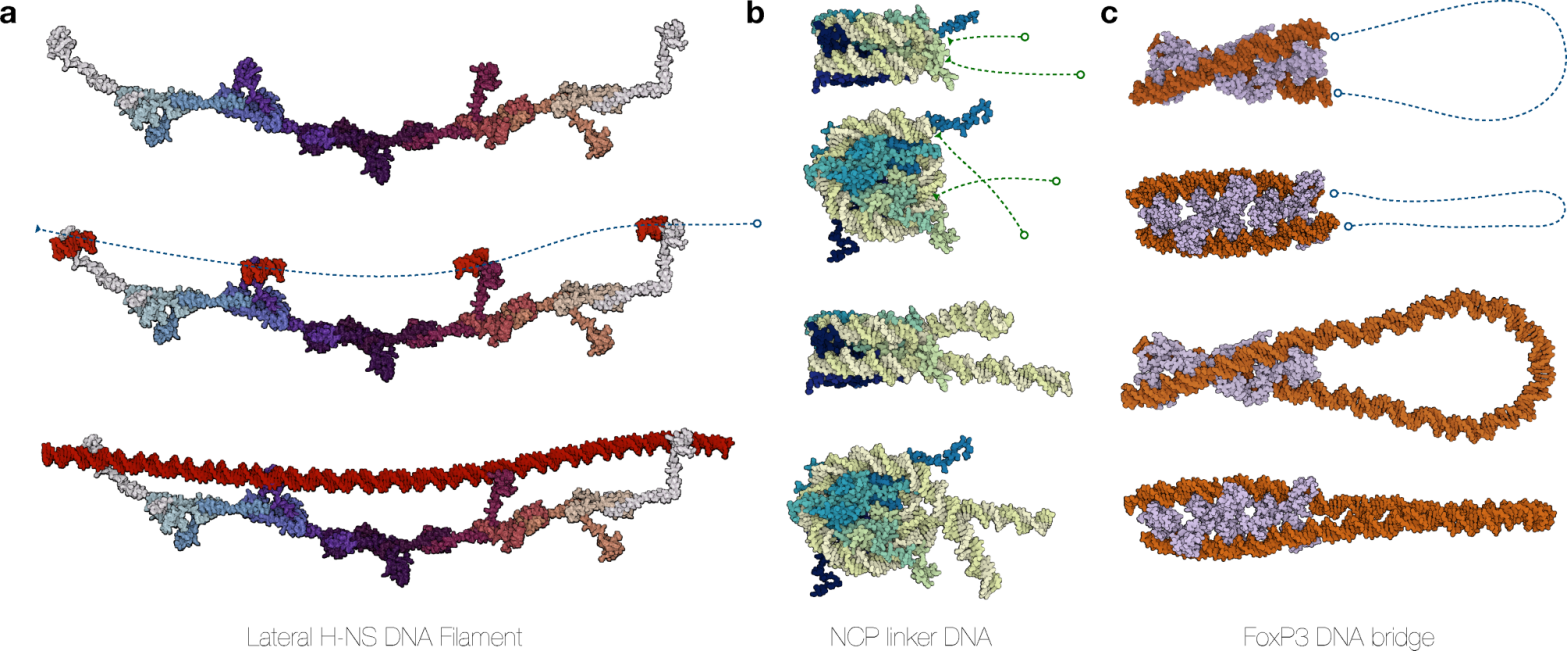
**A**) Constructing a continuous DNA strand along the H-NS filament. The process begins with an H-NS filament consisting of 12 subunits [65,66]. Next, four DBDs positioned along one side of the filament are selected, and a 12 bp DNA strand is superimposed onto these domains using structural data from the H-NS-DNA complex (PDB: 1HNS [67]) [68,69]. The DNA oligomers are shown in red. Finally, the reference frames of the four DNA oligomers are used as control points to generate a new DNA strand that spans the entire length of the H-NS filament. (**B**) Nucleosome (PDB: 1KX5 [48]) extended with 36 bp linker DNA in both the 5′ and 3′ direction. The extended DNA sequences are energy-minimized using the MC module. (**C**) Higher-order FoxP3 multimer that forms a ladder-like bridge to DNA (PDB: 8SRP) [70]. Here, we show how the bridged DNA ends can be connected with 200 bp that are minimized using the .minimize() function, creating a DNA loop held together by the FoxP3 ladder. Molecular representations are visualized with Mol* Viewer [46].

The ability to construct DNA–protein assemblies demonstrates MDNA‘s utility in enabling advanced modeling scenarios. Case studies include integration of DNA strands with H-NS filaments, extension of nucleosome-bound DNA to linker regions, and the creation of protein-mediated DNA loops. These examples demonstrate the utility of the toolkit in the construction of biologically relevant systems and bridge the gap between structural data and computational modeling studies. In the first example (Fig. 3A), the .make() function is used to create a continuous DNA strand along an H-NS filament. Starting with a filament of 12 H-NS subunits [65], four DNA-binding domains (DBDs) are selected, and a 12 bp DNA strand is superimposed onto these domains [68,69]. See Supplementary Section S6 and Supplementary Fig. S3 for details on how the H-NS filament was constructed. This method allows for the generation of a new DNA strand that spans the entire length of the filament, showcasing the tool’s ability to construct specified DNA shapes from scratch using protein filaments as scaffolds.

In the second example (Fig. 3B), the .extend() function is employed to extend a DNA sequence, adding 36 bp linker DNA to a nucleosome in both the 5′ and 3′ directions. This function accepts several parameters that allow precise control over the extension of the DNA strand: Users can specify the number of base pairs (n_bp) or provide a DNA sequence for the extension. The forward boolean parameter determines the direction of the extension. Users can also choose a time frame (frame) and shape (control_points) to define the new DNA shape. Users can determine if the terminal base pairs of the original structure are included or excluded from the minimization procedure (margin); increasing this parameter includes more bases of the original strand to also be minimized. This feature enables smoother transitions between the original DNA and the newly generated DNA.

The .connect() function is able to create a single DNA structure from two separate segments, and similarly to .extend() includes the arguments n_bp, frame, control_points, and margin. The function interpolates a straight line between the ends and distributes the optimal number of base pairs to achieve a neutral twist. The function requires two Nucleic objects and optional parameters to generate the DNA structure. In the final example (Fig. 3C), the .connect() function is used to link two separate DNA structures by creating a strand between the 3′ end of the first and the 5′ end of the second. This example illustrates how the function can generate a continuous DNA loop held together by the FoxP3 protein, which forms a ladder-like bridge between the two DNA ends [76,77]. Together, the functions .extend() and .connect() further provide additional features, allowing the creation and manipulation of complex DNA structures with precision.

## Conclusion

MDNA supports molecular simulations by providing atomic-resolution structural modeling of dsDNA in diverse shapes and compositions, including DNA–protein assemblies. By facilitating precise structural modeling of DNA at atomic resolution, MDNA contributes to improving our understanding of DNA dynamics and interactions in complex biological systems. The integration of structure generation, editing, and analysis tools into a single platform provides researchers with a streamlined workflow to address challenging questions in structural biology and molecular simulation. Its strength lies in a spline mapping protocol that enables the construction of template DNA structures in arbitrary shapes. In addition, MDNA facilitates the generation of diverse configurations supported by a MC minimization based on established DNA elastic models, including those incorporating non-canonical bases, WCF-to-HG transitions, and specific methylation patterns.

The case studies presented in this work, including the construction of DNA strands along an H-NS protein filament and the extension of nucleosome DNA, highlight the capability of MDNA to construct large protein–DNA complexes. By enabling the generation of initial atomic-resolution configurations tailored to specific experimental, theoretical, and topological needs, MDNA addresses a gap in existing tools for molecular simulations. In addition to its structure-generation capabilities, MDNA integrates various analysis tools within a unified Python-based ecosystem [78]. The integration of geometric analysis modules based on the rigid body formalism in the toolkit [13] further improves the utility to understand the structural dynamics and interactions of DNA. By incorporating libraries such as MDTraj and OpenMM [42,47], MDNA consolidates structure generation, simulation, and analysis into a streamlined workflow, reducing dependence on separate software platforms.

While MDNA can generate customizable structures and integrates analysis tools, some limitations present opportunities for future improvement. The lack of direct support for single-stranded DNA (ssDNA) structures restricts its applicability to processes such as replication, transcription, certain DNA repair pathways, and RNA modeling, which often involve hybrid or single-stranded configurations. Although ssDNA can be approximated by modifying the twist and removing the antistrand, a dedicated module for ssDNA and RNA generation would greatly enhance the versatility of the toolkit, particularly for studying hybrid RNA-DNA structures and RNA folding dynamics.Within its current scope, MDNA is designed for continuous double-stranded helices and does not explicitly represent branched architectures or single-stranded regions. Addressing these features, which are central to many DNA nanotechnology applications, will be an important focus of future work.

The MC-based structure relaxation, while effective, is the only optimization tool integrated into MDNA; other methods, such as steepest descent, would improve the flexibility of the toolkit. Additionally, the simulation of structures with the sequence library relies on existing force field parameters, which may not be available or adequately parameterized for non-canonical bases and modified nucleobases. These non-canonical bases are also incompatible with minimization using the MC model. MDNA is not yet optimized for kilobase (kb) length DNA structures; improving scalability for larger systems is a feasible and promising future direction. The optional nature of the minimization is advantageous for certain applications, as MDNA allows users to specify initially energetically unfavorable configurations, such as very tight knots or tiny minicircles. This flexibility cautions that initial structures should be used for qualitative analysis, as they may be non-physical depending on the choice of control points and may deviate from physical realism without further refinement. Future updates could also explore the integration of enhanced sampling methods, such as OpenPathSampling [79,79] or Plumed [80] workflows, to optimize starting structures and analyze free-energy landscapes in high-throughput scenarios.

In conclusion, MDNA is a versatile tool for the generation of dsDNA structures that offers insight into various dsDNA architectures and supports the setup of molecular dynamics simulations for DNA and protein–DNA complexes. The comprehensive features and integration with existing MD tools make MDNA a useful resource. Future updates will focus on expanding the support for the sequence library, introducing ssDNA and RNA functionality, and improving optimization algorithms. To support users at various levels of molecular modeling, MDNA is complemented by tutorials and demos. These resources improve accessibility for novice and experienced users, providing a starting point for educational applications such as workshops or classroom demonstrations.

## Supplementary Material

gkag549_Supplemental_File

## Data Availability

The MDNA source code is archived on Figshare and is publicly accessible via the following DOI: https://doi.org/10.6084/m9.figshare.31436725. In addition, the Figshare entry is linked to the GitHub repository to ensure long term preservation, version transparency, and reproducibility.
