# Supplementary material for: MDNA : a software module for DNA structure generation and analysis
Source: Nucleic Acids Res. 2026 Jun 2;54(10):gkag549. doi: 10.1093/nar/gkag549 (PMC13227108; doi:10.1093/nar/gkag549)
Supplement: gkag549_Supplemental_File [file gkag549_supplemental_file.pdf]

Supplementary Material for

MDNA: a software module for DNA structure generation and analysis

Thor van Heesch, Enrico Skoruppa, Peter G. Bolhuis, Helmut Schiessel, Jocelyne Vreede

Contents

S1 Frame Generation Algorithm **2**

S2 Back Mapping of Base Pairs **3**

S3 Monte Carlo Minimization **4**

S4 Calculation of Rigid Parameters **5**

S5 Calculation of Linking Number **9**

S6 Process of H-NS filament construction **10**

S7 Molecular Simulations Methods of Nucleosome and H-NS dimers **11**

S1 Frame Generation Algorithm

This section explains how to compute coordinate frames along a spline. A related problem in mathematics and computer graphics is the generation of coordinate frames with minimal torsion along a spline [1]. The solution involves initializing a coordinate frame at the beginning of the curve and incrementally propagating this frame along the spline [1]. This method relies only on the first derivative of the curve to compute subsequent frames and avoids problems with inflection points and torsion by maintaining continuity based on the previous frame. The process begins with the generation of a space curve that serves as the helical axis of DNA. The next step involves computing coordinate frames along equidistantly spaced points on the curve to guide the placement of the DNA's base pairs. Finally, a twist can be introduced to obtain a helical structure.

Given a set of control points $\mathbf{P} = P_1, P_2, \ldots, P_n$ in $\mathbb{R}^3$, a spline curve $S : [0,1] \to \mathbb{R}^3$ is defined as a piecewise polynomial function that smoothly interpolates through these points. The spline is initially defined in its native B-spline parameter domain and subsequently reparameterized by the arc length. The parameter $u \in [0,1]$, then represents the normalized arc length along the curve. For a cubic B-spline, the spline curve $S(u)$ as a weighted sum of control points P_i at any point u can be expressed as:

$$S(u) = \sum_{i=0}^{n} \mathbf{P}_i N_{i,k}(u) \tag{1}$$

where $N_{i,k}(u)$ are the basis functions of the B-spline with degree k, and $\mathbf{P}_i$ are the control points [2]. The degree is set by default to $k = 3$.

The procedural generation of DNA structures involves computing coordinate frames along $S(u)$, beginning with the computation of an initial frame followed by the propagation of this frame along the spline [1]. The goal is to construct an orthonormal basis defined by T, N, and B, which establishes a positively oriented moving frame at each curve point.

The initial tangent vector T_0 is calculated as the normalized first derivative of $S(u)$ at the starting point, indicating the direction of the curve:

$$T_0 = \frac{S'(u_0)}{\|S'(u_0)\|}. \tag{2}$$

The initial normal vector N_0 is determined by constructing a vector orthogonal to both T_0 and a predefined *reference* vector $\mathbf{v}_{ref}$, typically initialized as $[0,0,1]^T$:

$$N_0 = \frac{T_0 \times \mathbf{v}_{ref}}{\|T_0 \times \mathbf{v}_{ref}\|}. \tag{3}$$

If T_0 is parallel to $\mathbf{v}_{ref}$, an alternate vector is selected to ensure a non-zero cross product.

$$N_0 = \frac{T_0 \times \mathbf{v}_{ref}}{\|T_0 \times \mathbf{v}_{ref}\|}. \tag{4}$$

The initial binormal vector B_0 is calculated as the cross product of T_0 and N_0:

$$B_0 = T_0 \times N_0. \tag{5}$$

For each subsequent point u_i along the spline, the tangent vector T_i at u_i is computed as the normalized derivative of $S(u)$:

$$T_i = \frac{S'(u_i)}{\|S'(u_i)\|}. \tag{6}$$

The axis of rotation $\mathbf{a}_{i-1,i}$ is determined as the normalized cross product of T_{i-1} and T_i, indicating the direction to rotate N_{i-1} to compute N_i :

$$\mathbf{a}_{i-1,i} = \frac{T_{i-1} \times T_i}{\|T_{i-1} \times T_i\|}. \tag{7}$$

If the norm of $\mathbf{a}_{i-1,i}$ is very small (indicating negligible rotation), N_i is set to N_{i-1} to avoid discontinuity of the minimal torsion in the reference frames:

$$N_i = N_{i-1} \tag{8}$$

Otherwise, the angle of rotation $\theta_{i-1,i}$ is computed using the dot product of T_{i-1} and T_i :

$$\theta_{i-1,i} = \arccos\left(\frac{T_{i-1} \cdot T_i}{\|T_{i-1}\|\|T_i\|}\right) \tag{9}$$

Using the computed axis $\mathbf{a}_{i-1,i}$ and angle $\theta_{i-1,i}$, N_i is obtained by rotating N_{i-1} around $\mathbf{a}_{i-1,i}$. Finally, at u_i the binormal vector B_i is computed as:

$$B_i = T_i \times N_i. \tag{10}$$

The result is a twist free geometry described by an orthonormal coordinate system at a point on a surface or curve, consisting of tangent, normal, and binormal vectors that describe local geometric properties. A practical implementation of constructing and evaluating B-splines and their derivatives is provided by the `scipy` library [3].

Note that the more conventional approach using Frenet-Serret frames encounters difficulties at inflection points, where the second derivative of the curve is zero [4]. At these points, the normal and binormal vectors become undefined, leading to discontinuities and sudden inversions in the orientation of the coordinate frames. Although some approaches attempt to address this by introducing a fixed reference vector within the global coordinate system, these can still result in unwanted twisting at inflection points or regions where the curve aligns with the reference vector.

The spline generation and frame computation are the basis of our method for the procedural generation of DNA structures. To create a collection of reference frames that describes DNA as a space curve, we can introduce a helical twist by simply rotating the consecutive basis vectors along the base of $T(u)$ by the number of base pairs per turn (typically 10.5 bp). Alternatively, we can locally adjust the twist by specifying a custom twist value for a defined range of base pairs. The parameter u is evenly distributed along the spline, with all segments having the same size, d, which by default corresponds to an increase value of 0.34 nm. The distribution of frames is determined by parameterizing the arc length of the spline. The goal is to distribute n points along the spline, with fixed positions at both ends and a default spacing of 0.34 nm between each point. Due to the end constraints, the spacing of the last segment may be slightly larger than or less than 0.34 nm. To mitigate this, the total difference across the complete sequence is evenly distributed.

S2 Back Mapping of Base Pairs

The methodology for back mapping base pairs uses the DNA's reference frame, explained in Section S1, as a template to construct the atomic coordinates. The back mapping of the frames to the atomic coordinates starts with determining the sequence from the generated frames. After the sequence is set, the MDTraj library is used for the initialization of the trajectory, which contains the xyz coordinates and the topology of the system [5].

Initialization of the trajectory involves the generation of dummy coordinates for the sequence, which are idealized placeholder positions that will subsequently be transformed to match the spline geometry, and the use of the Tsukuba convention [6] to add a reference base frame to each base, using ideal forms for the initial structure of the bases. Subsequently, for the antisense chain, complementary bases are identified for positions from N to $2N-1$, and their coordinates undergo a transformation to ensure proper base pairing. This transformation involves rotating and flipping the coordinates of each complementary base by 180 degrees around the x-axis, which

lies in the plane of the base. This procedure guarantees that the first base of the anti-sense chain complements the sense strand's first base. The topology construction adheres to the progression from 5' to 3' for the leading chain with the anti-sense chain in reverse orientation. For circular DNA structures, we ensure the connection of terminal ends in their respective chains.

Following the preliminary construction of the DNA trajectory and topology with dummy coordinates, we compute the mean reference frames for the base pairs as described in Section S4. This step allows for the subsequent update of coordinates based on the reference frame derived from a spline. Let $\mathbf{O}_{\text{dummy}}$ and $\mathbf{O}_{\text{new}}$ denote the origins of the dummy and the new frames, respectively, and $\mathbf{B}_{\text{dummy}}$ and $\mathbf{B}_{\text{new}}$ represent the bases of these frames. Transformation from the old frame to the new frame involves calculating the rotation $\mathbf{R}$ and translation $\mathbf{T}$ matrices. The rotation matrix $\mathbf{R}$ is obtained by solving $\mathbf{B}_{\text{new}} = \mathbf{R}\mathbf{B}_{\text{dummy}}$, and the translation is determined by $\mathbf{T} = \mathbf{O}_{new} - \mathbf{O}_{\text{dummy}}$. These transformations are then applied to the dummy coordinates, finalizing the structural model with $\mathbf{X}_{\text{new}} = \mathbf{R}\mathbf{X}_{\text{dummy}} + \mathbf{T}$, where $\mathbf{X}_{\text{dummy}}$ and $\mathbf{X}_{new}$ denote the sets of old and updated coordinates, respectively.

S3 Monte Carlo Minimization

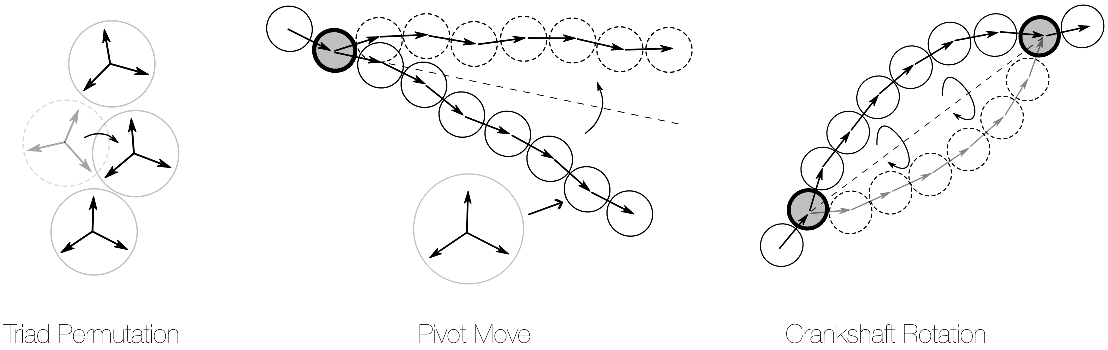

Supplementary Figure S1: The representation of moves used in the DNA triad model for generating new configurations using the MC method is illustrated from left to right as follows: single triad permutation, pivot move, and crankshaft rotation.

We provide energy minimization and thermalization of the generated DNA structures in the form of Monte Carlo (MC) sampling based on the rigid base pair framework [7, 8]. Using the procedure described in Supplementary Section S1, DNA molecules are mapped onto a sequence of triads. Relative orientations and positions of neighboring triads are parametrized in terms of the 3 rotational degrees of freedom (tilt, roll, and twist) and 3 translational degrees of freedom (shift, slide, and rise) that are condensed into junction vectors $\mathbf{X}_i \in \mathbb{R}^6$. These junction vectors correspond to the inter-base pair step parameters (shift, slide, rise, tilt, roll, twist), see Supplementary Section S4, then decomposed into static and dynamic components

$$\mathbf{X}_i = \mathbf{X}_{0,i} + \mathbf{X}_{\Delta,i}, \tag{11}$$

where the static components $\mathbf{X}_{0,i}$ specify the ground state of the molecule. The most important components of this vector are the intrinsic twist, reflecting the helicity of the molecule, and the intrinsic rise that determines the contour length per step of base pair. Fluctuations around this ground state are specified by an elastic Hamiltonian $\mathcal{H}(\mathbf{X}_\Delta)$ that penalizes deformations with the harmonic energy [9, 10, 7, 11]

$$\frac{\mathcal{H}(\mathbf{X}_\Delta)}{k_{\text{B}}T} = \frac{1}{2}\sum_i \mathbf{X}_{\Delta,i}^T M_{\mathbf{X}_i} \mathbf{X}_{\Delta,i}, \tag{12}$$

where $M_{\mathbf{X}_i} \in \mathbb{R}^{6\times 6}$ are sequence-dependent stiffness matrices and the summation extends to all junction vectors. Finally, we denote k_B as the Boltzmann constant and T as the temperature. At present, stiffness matrices and ground states are taken from atomistic simulations [10] or crystallographic data [9]. In this parametrization, the elastic energy is assumed to be local, i.e., fluctuations at each base pair step are decoupled from neighboring steps. Work of the last decade suggests this locality be insufficient to capture detailed length-scale dependent elastic features [12, 13, 14]. Parameters derived from more recent models (e.g., cgNA+ [15]) indeed provide a more general elastic description featuring non-local couplings. To thermalize and minimize DNA structures as initial configurations for Molecular Dynamics simulations, the local description is deemed sufficient.

Steric hindrance and screened electrostatic repulsion are modeled using hard-sphere potentials with effective diameters d_{EV}, depending on the ionic conditions of the experiment [16]. At a concentration of 150 mM univalent salt, an effective diameter of $d_{EV} = 4.0$ nm was found to agree well with the experimental single-molecule measurements [17]. Configurations are progressively generated with a series of permutation moves. These include pivot, crankshaft, and single-triad permutation (rotation and translation) moves [17, 18], see Supplementary Figure S1 for an illustration of these MC moves. The type of move and the number of triads involved are chosen at random. Rejection and acceptance are based on the Metropolis criterion.

Convergence during Monte Carlo minimization is assessed using automated equilibration criteria rather than by enforcing a predefined target state. In `MDNA`, equilibration is monitored through the evolution of an observable such as the total elastic energy or writhe depending on the protocol choice. Two approaches are available. In the first, the simulation is divided into consecutive windows of fixed length, and convergence is inferred once the window-averaged observable no longer shows a systematic upward or downward trend, remaining within previously observed extrema for a specified number of windows. In the second approach, the decay of the observable is explicitly fitted to an exponential function, yielding a characteristic decay scale in terms of Monte Carlo cycles. The system is considered equilibrated once the simulation has exceeded a fixed multiple of this characteristic scale (the default is 6). Both criteria assume a monotonic relaxation toward equilibrium and may fail in the presence of slow barrier-crossing events. In such cases, equilibration can be performed manually, with the stopping point chosen by the user.

S4 Calculation of Rigid Parameters

In this work, we distinguish between curve-adapted frames generated along the spline, and base-attached reference frames constructed from atomic coordinates. The spline frames describe the global helical axis geometry, whereas the base reference frames are rigid-body frames attached to individual nucleobases. The Cambridge convention established the rigid body parameters for the geometry of nucleic acids but does not specify the precise construction of the reference frame [6]. Tools such as 3DNA [19] and Curves+ [20] demonstrate the effectiveness of rigid body models, serving as benchmarks for the rigid base definitions, with their similarities and differences extensively described elsewhere [21]. Rigid body models treat each DNA base as a single entity. Each base b is assigned a reference frame for precise spatial positioning and orientation. The frame consists of a reference point, $\mathbf{r_b}$, and a frame, $\mathbf{B_b}$, consisting of an orthonormal triad of vectors pointing towards the major groove, backbones, and directing perpendicularly to the base plane. The collection of these base-attached reference frames defines the rigid-body representation of the DNA molecule. Transformations between these base reference frames distinguish DNA conformations through rotational matrices and translation vectors that define the intra- and inter-base pair coordinates. For intra-base pair movements, they detail rotations (buckle, propeller, opening) and translations (shear, stretch, stagger), capturing the nuances of individual base pair positioning. On the other hand, for inter-base pair steps, they define rotations (tilt,

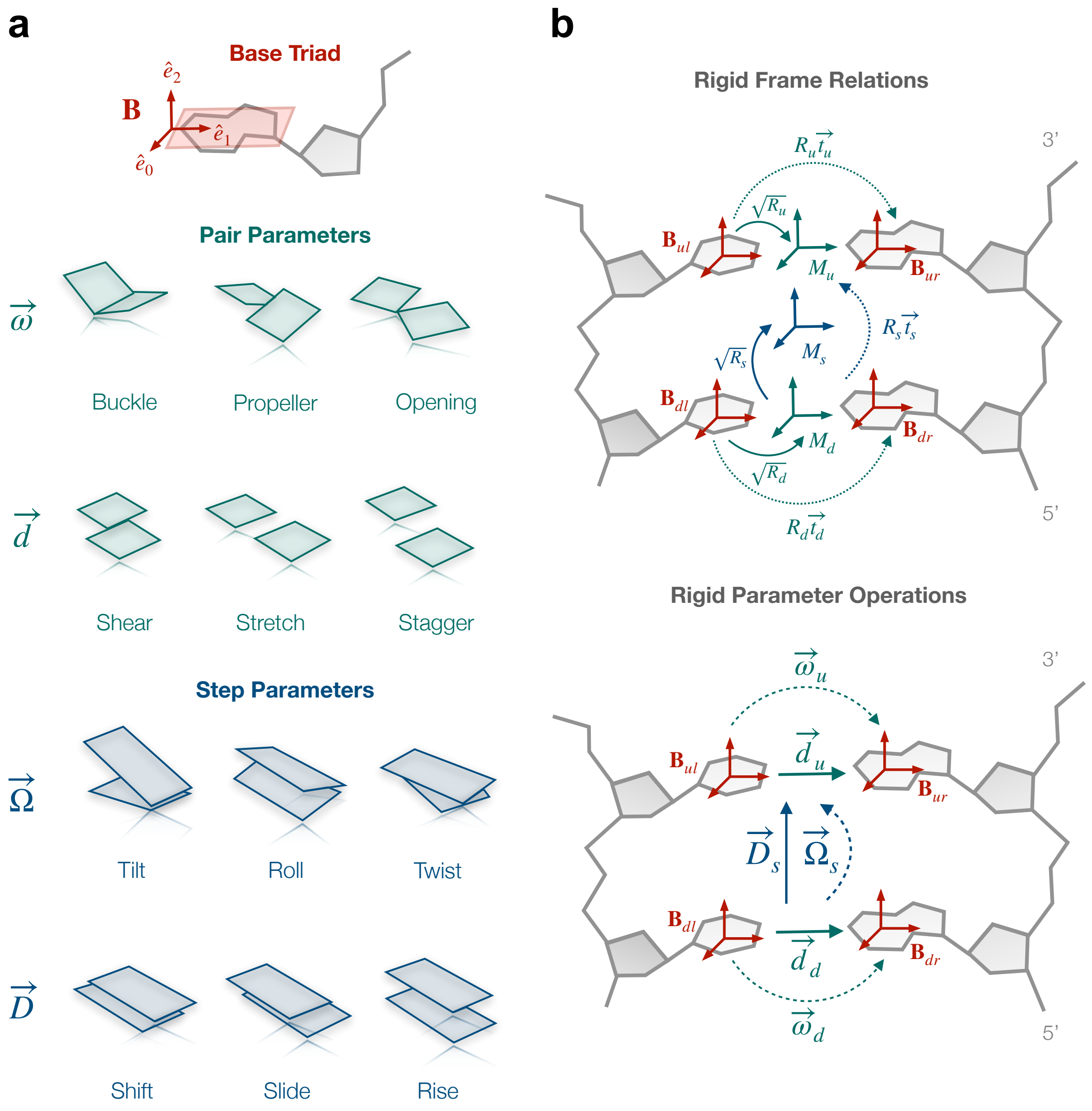

Supplementary Figure S2: Top: Illustration of the base triad, pair (intra-base) and step (inter-base) parameters and the geometric operations used to obtain rigid-body coordinates. **a)** graphical representation of the unit vectors, $\hat{e}_i$, forming a single base triad, $\mathbf{B}$, attached to one nucleobase. Middle: intra-base pair rotational (buckle, propeller, opening) and translational (shear, stretch, stagger) pair parameters. Bottom: inter-base pair step rotational (tilt, roll, twist) and translational (shift, slide, rise) step parameters. Bases and base pairs are represented as cuboids. **b)** Top: geometric relations between upper left and right complementary base frames $\mathbf{B}_{ul}$ and $\mathbf{B}_{ur}$ and downwards complementary frames $\mathbf{B}_{dl}$ and $\mathbf{B}_{dr}$, their associated midframe $\mathbf{M}_u$, and the step midframe $\mathbf{M}_s$. Intra-base pair coordinates describe the rigid-body transformation between complementary base triads, while inter-base pair step coordinates describe the transformation between successive base-pair midframes ($\mathbf{M}_u$ and $\mathbf{M}_d$). Bottom: extraction of translational ($\vec{d}, \vec{D}$) and rotational ($\vec{\omega}, \vec{\Omega}$) rigid-body parameters from the corresponding frame transformations.

roll, twist) and translations (shift, slide, rise), providing the spatial and rotational relationships between adjacent base pairs, also known as step parameters.

To obtain the rigid base parameters we followed the implementation as described in Curves+ [20] and Petkevičiūtė et al. [22]. For each base, we construct a reference frame $\{\mathbf{B}_b, \mathbf{r}_b\}$ consisting of an orthonormal triad of unit vectors $\mathbf{B}_b = \{\hat{e}_0, \hat{e}_1, \hat{e}_2\}$ and their origin $\mathbf{r}_b$. In this setup, the

base triad vector $\hat{e}_0$ points to the major groove, $\hat{e}_1$ connects the backbones, and $\hat{e}_2$ is normal to the base plane. See the top panel of Supplementary Figure S2-a for a cartoon representation of a single base triad.

To parameterize translations and rotations connecting two complementary base frames on opposite strands of a DNA molecule, one uses the rigid base coordinates $\vec{d}$ (a vector whose components are referred to as shear, stretch, and stagger in the DNA literature [23]) and $\vec{\omega}$ (buckle, propeller, and opening). The coordinates $\vec{D}$ (shift, slide, and rise) and $\vec{\Omega}$ (tilt, roll, and twist) parameterize translations and rotations between two consecutive base pairs. See Supplementary Figure S2-a for a visual representation of the rigid base parameters.

To obtain the translational and rotational parameters for each base and base pair, we start with the transformation between two opposing base frames ($\mathbf{B}_{ur}$ and $\mathbf{B}_{ul}$), characterized by a rotation matrix $\mathbf{R}_u$ and a translation vector $\vec{t}_u$:

$$\mathbf{R}_u = \mathbf{B}_{ur}\mathbf{B}_{ul}^T, \quad \vec{t}_u = \mathbf{r}_{ur} - \mathbf{r}_{ul} \tag{13}$$

These transformations are expressed in a midframe $\mathbf{M}_u$, which is determined by rotating the vectors in $\mathbf{B}_{ul}$ by half the rotation angle of $\mathbf{R}_u$. Next, we compute the angle $\Theta(\mathbf{R})$ and the axis $\hat{K}(\mathbf{R})$ of rotation.

The angle $\Theta(\mathbf{R})$ and the axis $\hat{K}(\mathbf{R})$ of rotation are given by:

$$\Theta(\mathbf{R}) = \arccos\left(\frac{\mathrm{Tr}(\mathbf{R}) - 1}{2}\right) \tag{14}$$

$$\hat{K}(\mathbf{R}) = \frac{1}{2\sin\Theta(\mathbf{R})}\begin{bmatrix} R_{23} - R_{32} \\ R_{31} - R_{13} \\ R_{12} - R_{21} \end{bmatrix} \tag{15}$$

The rotational and translational parameters of the base pair are as follows:

$$\vec{\omega}_u = \Theta(\mathbf{R}_u)\left(\hat{K}(\mathbf{R}_u)^T \cdot \mathbf{M}_u\right), \quad \vec{d}_u = \vec{t}_u^{\,T} \cdot \mathbf{M}_u \tag{16}$$

The process repeats for successive base pairs $\mathbf{B}_{dr}$ and $\mathbf{B}_{dl}$. After this, we can use the respective mid frames of the base pairs, $\mathbf{M}_u$ and $\mathbf{M}_d$, where the transformation between pairs involves a new rotation $\mathbf{R}_s$ and translation $\vec{t}_s$:

$$\mathbf{R}_s = \mathbf{M}_u\mathbf{M}_d^T, \quad \vec{t}_s = \frac{1}{2}(\mathbf{r}_{ur} + \mathbf{r}_{ul}) - \frac{1}{2}(\mathbf{r}_{dr} + \mathbf{r}_{dl}) \tag{17}$$

The midframe $\mathbf{M}_s$ for this step is defined analogously:

$$\mathbf{M}_s = \sqrt{\mathbf{R}_s}\mathbf{M}_d = \sqrt{\mathbf{R}_s^T}\mathbf{M}_u \tag{18}$$

Finally, the transformations are expressed in this base step midframe, with the corresponding rotational and translational variables:

$$\vec{\Omega}_s = \Theta(\mathbf{R}_s)\left(\hat{K}(\mathbf{R}_s)^T \cdot \mathbf{M}_s\right), \quad \vec{D}_s = \vec{t}_s^{\,T} \cdot \mathbf{M}_s \tag{19}$$

Supplementary Figure S2-b shows a diagram of both the relations (top) and the operations (bottom) to obtain the rigid base parameters.

Supplementary Table S1: Glossary of coordinate systems and rigid-base geometry in DNA modeling.

**Term**	**Definition**
**I. General Coordinate Systems**	
Reference frame	A coordinate system $\{\mathbf{B}, \mathbf{r}\}$, where $\mathbf{r} \in \mathbb{R}^3$ is the origin or reference point and $\mathbf{B} = \{\hat{e}_0, \hat{e}_1, \hat{e}_2\}$ is an orthonormal basis defining orientation also referred to as a triad.
Frame	A coordinate system defined by an origin and an orthonormal triad. Specific instances include base frames, mid frames, and curve-adapted frames.
Reference point	The origin $\mathbf{r}$ of a frame.
Triad	An ordered orthonormal basis $\{\hat{e}_0, \hat{e}_1, \hat{e}_2\}$ in $\mathbb{R}^3$, defining a right-handed local coordinate system.
Orthonormal	A triad satisfying $\hat{e}_i \cdot \hat{e}_j = \delta_{ij}$ and $\hat{e}_0 \times \hat{e}_1 = \hat{e}_2$.
Unit vector	A vector of magnitude one, $\|\hat{e}\| = 1$.
Rotation matrix	A matrix $\mathbf{R} \in SO(3)$ whose columns form a right-handed orthonormal triad and it represents a change of orientation between frames.
Mean reference frame	A frame constructed from two frames by applying half of the relative rotation, defined via the matrix square root of the relative rotation, to one of them. Translational components are subsequently expressed in this midframe.
Base frame	A local right-handed orthonormal frame $\{\mathbf{B}_b, \mathbf{r}_b\}$ attached to a base. The triad $\mathbf{B}_b = \{\hat{e}_0, \hat{e}_1, \hat{e}_2\}$ is defined such that $\hat{e}_0$ points toward the major groove, $\hat{e}_1$ connects the backbones, and $\hat{e}_2$ is normal to the base plane.
**II. Base and Curve-Adapted Frames**	
Base plane	The geometric plane fitted through selected base atoms, characterized by a unit normal vector $\hat{\mathbf{n}}$.
Base triad	An orthonormal triad $\mathbf{B}_b = \{\hat{e}_0, \hat{e}_1, \hat{e}_2\}$ attached to a base or base pair rigid body.
Base triad vector	One of the unit vectors $\hat{e}_i$ belonging to a base triad.
**III. Rigid-Body Description**	
Rigid-body coordinates	A six-component vector composed of three translational and three rotational parameters derived from a relative rigid-body transformation in $SE(3)$. The translational part $\mathbf{r} \in \mathbb{R}^3$ represents the displacement between frame origins, while the rotational part is obtained from the associated rotation matrix $\mathbf{R} \in SO(3)$ using an axis–angle representation.
Rigid-body parameters	Six relative parameters connecting two frames: three translations and three rotations.
Translation vector	A displacement vector $\vec{d} = (d_1, d_2, d_3)$ expressed in a chosen frame, obtained from the difference of frame origins and projected into the appropriate midframe for parameter extraction.

Continued on next page

Term	Definition
Rotation vector	An angular displacement vector $\vec{\omega} = (\omega_1, \omega_2, \omega_3)$ parameterizing a rotation in $\mathbb{R}^3$ using an axis–angle representation.
**IV. Intra-Base Pair Geometry**	
Intra-base pair	Relative rigid-body parameters between two complementary base frames, obtained from their relative transformation and expressed in the corresponding base-pair midframe.
Intra-base pair coordinates	Relative rigid-body parameters $(\vec{d}, \vec{\omega})$ obtained from the relative transformation between complementary base frames and expressed in the base-pair midframe.<br>Translations: shear, stretch, stagger.<br>Rotations: buckle, propeller, opening.
**V. Inter-Base Pair Geometry**	
Inter-base pair	Relative rigid-body parameters between two consecutive base-pair midframes, obtained from their relative transformation and expressed in the step midframe.
Inter-base pair coordinates	Relative rigid-body step parameters $(\vec{D}, \vec{\Omega})$ obtained from the transformation between consecutive base-pair midframes and expressed in the step midframe.<br>Translations: shift, slide, rise.<br>Rotations: tilt, roll, twist.
Inter-base pair step	The geometric transformation relating two consecutive base-pair frames.

S5 Calculation of Linking Number

Intuitively, the linking number Lk of two oriented closed curves $\mathcal{C}_1$ and $\mathcal{C}_2$ describes the frequency with which the curves wind around each other. Alternatively, it can be seen as the number of times one strand crosses any surface bounded by the other, formalized by Gauss's linking integral [24]:

$$\mathrm{Lk} = \frac{1}{4\pi} \oint_{\mathcal{C}_1} \oint_{\mathcal{C}_2} d\mathbf{r}_1 \times d\mathbf{r}_2 \cdot \frac{\mathbf{r}_1 - \mathbf{r}_2}{|\mathbf{r}_1 - \mathbf{r}_2|^3}, \tag{20}$$

where $\mathbf{r}_1$ and $\mathbf{r}_2$ are the points along the two curves $\mathcal{C}_1$ and $\mathcal{C}_2$, respectively.

In a relaxed dsDNA molecule, the right-handed helical structure introduces one link per helical repeat ($n_h = 10.5$ bp or $h = 3.57$ nm for B-DNA). For a molecule of length L this results in a relaxed state linking number of $Lk_0 = L/h$.

The sign of the linking depends on the directionality of the strand. For DNA, it is typically defined so that Lk is positive for right-handed helices and negative for left-handed ones. However, the linking number Lk may differ from Lk_0 when the helix is over- or under-twisted, leading to an excess linking number $\Delta Lk = Lk - Lk_0$.

The linking number is a conserved topological property that always assumes integer values. However, according to the Călugăreanu-White-Fuller theorem [25, 26, 27], the linking number of two parallel running space curves may be decomposed into two geometric quantities—twist and writhe—as

$$\mathrm{Lk} = \mathrm{Tw} + \mathrm{Wr}. \tag{21}$$

Twist indicates local winding of the two strands, whereas writhe indicates non-local coiling.

Twist, as a local property, involves a single integral over twist density, while writhe, a non-local property, requires a double integral akin to Gauss' integral. For any closed, smooth curve $\mathcal{C}$, the writhe Wr is given by:

$$\mathrm{Wr} = \frac{1}{4\pi} \int_{\mathcal{C}} \int_{\mathcal{C}} d\mathbf{r}_1 \times d\mathbf{r}_2 \cdot \frac{\mathbf{r}_1 - \mathbf{r}_2}{|\mathbf{r}_1 - \mathbf{r}_2|^3}. \tag{22}$$

where $\mathbf{r}_1$ and $\mathbf{r}_2$ are points on the curve $\mathcal{C}$.

This integral spans the center line curve $\mathcal{C}$ of the helix. Unlike linking, writhe is tied to a single space curve, reflecting the average signed crossings from all perspectives [28] and vanishing in fully planar curves or those with reflection symmetry.

In practice, we evaluate the linking number for a chain of straight segments, which naturally decomposes Gauss's linking integral into a double sum of pairwise components. Contributions to the linking number stemming from such pairs of straight segments may be evaluated analytically; for details, see [29] and Method 1b in Ref. [30]. Similarly, for the numerical evaluation of Eq. 22, the backbone is represented as a polygonal chain of straight segments and the writhe double integral is evaluated as an analytical sum over all segment pairs, following Method 1a of Ref. [30].

The superhelical density $\sigma \equiv \Delta\mathrm{Lk}/\mathrm{Lk}_0$ can be computed in post-processing from `MDNA` outputs once a relaxed linking number $\mathrm{Lk}_0 = N_{\mathrm{bp}}/n_h$ has been specified, where n_h is the helical repeat in base pairs per turn [17]. This quantity is both sequence- and model-dependent: the local helical repeat varies with dinucleotide composition [9], and coarse-grained or atomistic force fields may relax to helical repeats that differ from the canonical solution value of $n_h \approx 10.5$ bp/turn. For short constructs, these differences can shift σ by amounts comparable to physiological superhelical densities ($-0.06 \lesssim \sigma \lesssim -0.04$). We therefore recommend determining Lk_0 self-consistently from torsionally relaxed reference structures generated with the same model.

S6 Process of H-NS filament construction

H-NS (Histone-like nucleoid-structuring protein) is a bacterial DNA-binding protein that plays a crucial role in the organization of the nucleoid in gram-negative enterobacteria. The protein structures DNA by forming filaments along DNA duplexes, either by binding to two separate DNA duplexes or to adjacent sites on the same duplex [35, 36, 37, 38, 39, 40]. H-NS is made up of 137 amino acid residues, divided into two main domains: the oligomerization domain and the DNA binding domain (DBD). The oligomerization domain, which consists of residues 1-83, includes two key sites: the homodimerization site (s1) and the multimerization site (s2), which allow the formation of higher order structures [32]. The DBD, which spans residues 89-137, consists of an antiparallel β-sheet, an α helix, and a 3_{10} helix [41, 33]. NMR experiments on the full H-NS protein indicated that the oligomerization domain and DBD function independently of each other, with a flexible linker connecting the two domains [42].

Given the absence of detailed structural data for H-NS filaments, the following protocol outlines a computational approach to construct H-NS filaments using available structural data and a protocol outlined by Van der Valk et al. [43], see Supplementary Figure S3. To construct a full-length H-NS monomer we used residues 2–83, from *S. typhimurium* (PDB 3NR7 [32]) and the NMR structure of the C-terminal domain, residues 91–137 (PDB 2L93 [33]), while non-resolved sequences (residues 1 and 84–90) were modeled as a random coil. The structure of residues 2–83 of *S. typhimurium* H-NS also contained information on the dimerization sites. The coordinates of the DNA binding domain (DBD) of H-NS bound to the minor groove of a high-affinity 12-bp strand of dsDNA were obtained from earlier work [31]. Using these structures as templates, we combined 12 H-NS monomers into a multimer. To form the multimer, functional segments within the H-NS structure are identified: s1 (homodimerization domain), h3 (a helical region that potentially connects the s1 and s2 domains), s2 (multimerization domain), l2 (flexible linker

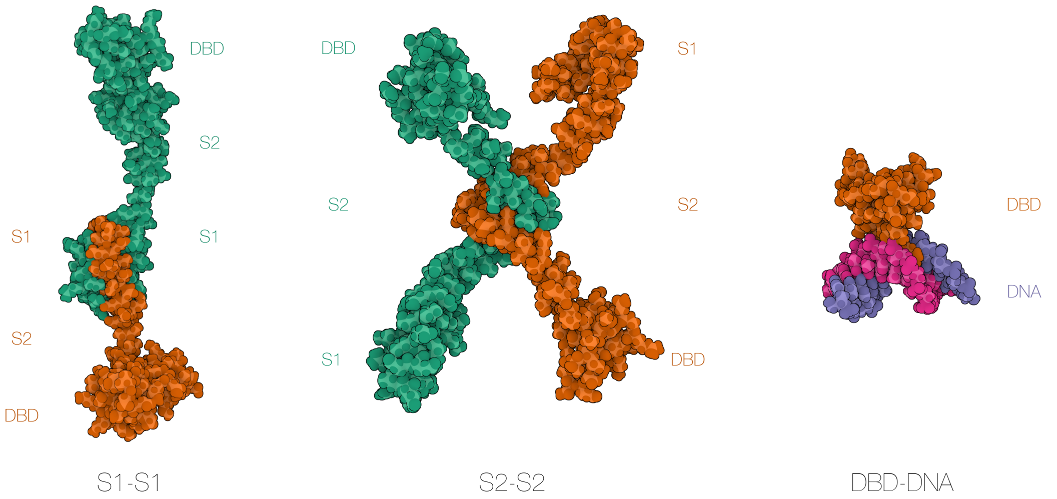

Supplementary Figure S3: H-NS systems used to generate lateral protein filament: the H-NS homodimer (s1s1 system), the multimerization dimeric unit (s2s2 system), and the DNA binding domain (DBD) DNA complex. Coordinates of the DNA binding domain (DBD) of H-NS bound to the minor groove of a high-affinity 12-bp strand of dsDNA were obtained from earlier work [31]. Systems s1s1 and s2s2 were constructed using two monomers. The monomers were constructed based (PDB 3NR7 [32] and PDB 2L93 [33]). Molecular representations are visualized with Mol* Viewer [34].

region), and DBD (DNA binding domain). To form the multimer, we also included two structures as a building block where two s1 segments and two s2 segments are non-covalently bound. The segments are aligned using superposition techniques, where the root mean squared deviation (RMSD) of atom selections based on the overlapping residues between segments is minimized using singular value decomposition (SVD). Following alignment, the structures are joined to form a continuous H-NS filament. During this assembly process, the residue numbering is adjusted to maintain chain continuity. The initial conformations of the segments are selected based on short molecular dynamics (MD) simulations of the s1s1 and s2s2 system (see Supplementary Figure S3) that ensure that the filament grows linearly, without intersecting with itself, causing overlap between the atomic positions. For computational details of the MD simulations, see section S7. The code to reproduce this protocol can be found on the documentation webpage.

S7 Molecular Simulations Methods of Nucleosome and H-NS dimers

Molecular dynamics (MD) simulations of the following systems have been performed using the protocol outlined below. The Nucleosome Core Particle with 147 bp DNA has been simulated for a duration of 250 ns for the analysis as shown in Section 2.4. For the construction of the H-NS filament as described in S6, the following MD simulations have been performed: For the s1s1 system, 15 runs of 62.5 ns starting from the same structural configuration were performed. Additionally, there are two longer runs of 100 ns each. These runs were initiated from a closed or open state characterized by the availability of the DNA binding domain (DBD) for DNA binding. For the s2s2 system, 12 simulations of 100 ns each were performed, all starting from the same initial structure.

The preparation of the systems for MD simulations consisted of placing the structures in a periodic dodecahedron box, with the box boundaries at least 1.0 nm from the system, followed by the addition of water molecules. To mimic experimental conditions [33] and neutralize the

system, we added 50 mM NaCl by replacing water molecules with ions. The interactions between atoms are described by the force field AMBER14sb-parmbsc1 [44, 45] in combination with the TIP3P water model [46]. We selected this particular force field because it covers the topologies for both amino acids and nucleotides and provides good representations of the static and dynamic properties of DNA under a wide range of conditions [45]. For non-bonded interactions, both van der Waals and electrostatic, we used a cut-off at 1.1 nm. Long-range electrostatic interactions were handled using the particle mesh Ewald method [47, 48] with a grid spacing of 0.12 nm. To remove unfavorable interactions, we performed energy minimization using steepest descent. By applying position restraints on the heavy atoms of the protein and DNA with a force constant in each direction of 1000 kJ/mol nm^2 and performing 0.1 ns of MD at a temperature of 298 K and a pressure of 1 bar, we relaxed the water and ions around the initial structures.

After preparation, we performed MD simulations varying initial conditions by assigning new random starting velocities drawn from the Maxwell-Boltzmann distribution at 298 K. All simulations were performed with GROMACS, version 2020.4 [49, 50] in a locally maintained cluster, with the leap-frog integration scheme and a time step of 2 fs, using LINCS [51] to constrain the proteins and SETTLE [52] to constrain the water bonds. All simulations were performed in the isothermal-isobaric ensemble at a pressure of 1 bar, using the v-rescale thermostat [53] and the isotropic Parrinello-Rahman barostat [54, 55].

References

[1] Jules Bloomenthal. Calculation of reference frames along a space curve. *Graphics Gems*, 1:567–571, 1990.

[2] Carl De Boor. *A practical guide to splines*, volume 27. Springer New York, 1978.

[3] Pauli Virtanen, Ralf Gommers, Travis E Oliphant, Matt Haberland, Tyler Reddy, David Cournapeau, Evgeni Burovski, Pearu Peterson, Warren Weckesser, Jonathan Bright, et al. SciPy 1.0: fundamental algorithms for scientific computing in Python. *Nature Methods*, 17(3):261–272, 2020.

[4] Andrew N Pressley. *Elementary differential geometry.* Springer Science & Business Media, 2010.

[5] Robert T McGibbon, Kyle A Beauchamp, Matthew P Harrigan, Christoph Klein, Jason M Swails, Carlos X Hernández, Christian R Schwantes, Lee-Ping Wang, Thomas J Lane, and Vijay S Pande. MDTraj: a modern open library for the analysis of molecular dynamics trajectories. *Biophysical Journal*, 109(8):1528–1532, 2015.

[6] Richard E Dickerson. Definitions and nomenclature of nucleic acid structure parameters. *Journal of Biomolecular Structure and Dynamics*, 6(4):627–634, 1989.

[7] F. Lankaš, O. Gonzalez, L. M. Heffler, G. Stoll, M. Moakher, and J. H. Maddocks. On the parameterization of rigid base and basepair models of DNA from molecular dynamics simulations. *Phys. Chem. Chem. Phys.*, 11(45):10565–10588, 2009.

[8] Behrouz Eslami-Mossallam, Raoul D. Schram, Marco Tompitak, John van Noort, and Helmut Schiessel. Multiplexing Genetic and Nucleosome Positioning Codes: A Computational Approach. *PLOS ONE*, 11(6):e0156905, 2016.

[9] W. K. Olson, A. A. Gorin, X. Lu, L. M. Hock, and V. B. Zhurkin. DNA sequence-dependent deformability deduced from protein–DNA crystal complexes. *Proc. Natl. Acad. Sci. U.S.A.*, 95:11163–11168, 1998.

[10] F. Lankaš, J. Šponer, J. Langowski, and T. E. Cheatham III. DNA basepair step deformability inferred from molecular dynamics simulations. *Biophys J.*, 85:2872–2883, 2003.

[11] Enrico Skoruppa and Helmut Schiessel. Systematic coarse-graining of sequence-dependent structure and elasticity of double-stranded DNA. *Physical Review Research*, 7(1):013044, 2025.

[12] Agnes Noy and Ramin Golestanian. Length scale dependence of DNA mechanical properties. *Phys. Rev. Lett.*, 109:228101, 2012.

[13] Enrico Skoruppa, Aderik Voorspoels, Jocelyne Vreede, and Enrico Carlon. Length-scale-dependent elasticity in DNA from coarse-grained and all-atom models. *Phys. Rev. E*, 103:042408, 2021.

[14] Midas Segers, Aderik Voorspoels, Takahiro Sakaue, and Enrico Carlon. Mechanical properties of nucleic acids and the non-local twistable wormlike chain model. *J. Chem. Phys.*, 156(23):234105, 06 2022.

[15] R. Sharma, A. S. Patelli, L. de Bruin, and J. H. Maddocks. cgNA+web: A Visual Interface to the cgNA+ Sequence-dependent Statistical Mechanics Model of Double-stranded Nucleic Acids. *J. Mol. Biol.*, 435(14):167978, 2023.

[16] Valentin V Rybenkov, Nicholas R Cozzarelli, and Alexander V Vologodskii. Probability of DNA knotting and the effective diameter of the DNA double helix. *Proc. Natl. Acad. Sci. USA*, 90(11):5307–5311, 1993.

[17] W. Vanderlinden, E. Skoruppa, P. J. Kolbeck, E. Carlon, and J. Lipfert. DNA fluctuations reveal the size and dynamics of topological domains. *PNAS Nexus*, 1(5):pgac268, 11 2022.

[18] E. Skoruppa and E. Carlon. Equilibrium fluctuations of DNA plectonemes. *Phys. Rev. E*, 106:024412, 2022.

[19] Xiang-Jun Lu and Wilma K Olson. 3DNA: a software package for the analysis, rebuilding and visualization of three-dimensional nucleic acid structures. *Nucleic Acids Research*, 31(17):5108–5121, 2003.

[20] Richard Lavery, MJHPD Moakher, John H Maddocks, D Petkeviciute, and Krystyna Zakrzewska. Conformational analysis of nucleic acids revisited: Curves+. *Nucleic Acids Research*, 37(17):5917–5929, 2009.

[21] Filip Lankas and T Schlick. Modelling nucleic acid structure and flexibility: from atomic to mesoscopic scale. *Innovations in Biomolecular Modeling and Simulations: Volume 2*, page 1, 2012.

[22] Daiva Petkeviciute. A DNA coarse-grain rigid base model and parameter estimation from molecular dynamics simulations. Technical report, EPFL, 2012.

[23] Wilma K Olson, Manju Bansal, Stephen K Burley, Richard E Dickerson, Mark Gerstein, Stephen C Harvey, Udo Heinemann, Xiang-Jun Lu, Stephen Neidle, Zippora Shakked, et al. A standard reference frame for the description of nucleic acid base-pair geometry. *Journal of Molecular Biology*, 313(1):229–237, 2001.

[24] Carl Friedrich Gauss, Carl Friedrich Gauss, and Königlichen Gesllschaft der Wissenschaften zu Göttingen. Zur mathematischen Theorie der electrodynamischen Wirkungen. *Werke: Fünfter Band*, pages 601–630, 1877.

[25] Georges Călugăreanu. Sur les classes d'isotopie des noeuds tridimensionnels et leurs invariants. *Czechoslovak Mathematical Journal*, 11(4):588–625, 1961.

[26] F Brock Fuller. The writhing number of a space curve. *Proceedings of the National Academy of Sciences*, 68(4):815–819, 1971.

[27] James Harris White. *Self-linking and the Gauss integral in higher dimensions.* University of Minnesota, 1968.

[28] Konstantin Klenin and Jörg Langowski. Computation of writhe in modeling of supercoiled DNA. *Biopolymers: Original Research on Biomolecules*, 54(5):307–317, 2000.

[29] A. V. Vologodskii, V. V. Anshelevich, A. V. Lukashin, and M. D. Frank-Kamenetskii. Statistical mechanics of supercoils and the torsional stiffness of the DNA double helix. *Nature*, 280(5720):294–298, 1979.

[30] Konstantin Klenin and Jörg Langowski. Computation of writhe in modeling of supercoiled DNA. *Biopolymers*, 54(5):307–317, 2000.

[31] Enrico Riccardi, Eva C Van Mastbergen, William Wiley Navarre, and Jocelyne Vreede. Predicting the mechanism and rate of H-NS binding to AT-rich DNA. *PLoS Computational Biology*, 15(3):e1006845, 2019.

[32] Stefan T Arold, Paul G Leonard, Gary N Parkinson, and John E Ladbury. H-NS forms a superhelical protein scaffold for DNA condensation. *Proceedings of the National Academy of Sciences*, 107(36):15728–15732, 2010.

[33] Blair RG Gordon, Yifei Li, Atina Cote, Matthew T Weirauch, Pengfei Ding, Timothy R Hughes, William Wiley Navarre, Bin Xia, and Jun Liu. Structural basis for recognition of AT-rich DNA by unrelated xenogeneic silencing proteins. *Proceedings of the National Academy of Sciences*, 108(26):10690–10695, 2011.

[34] David Sehnal, Sebastian Bittrich, Mandar Deshpande, Radka Svobodová, Karel Berka, Václav Bazgier, Sameer Velankar, Stephen K Burley, Jaroslav Koča, and Alexander S Rose. Mol* Viewer: modern web app for 3D visualization and analysis of large biomolecular structures. *Nucleic Acids Research*, 49(W1):W431–W437, 2021.

[35] M Falconl, MT Gualtierl, A La Teana, MA Losso, and CL Pon. Proteins from the prokaryotic nucleoid: primary and quaternary structure of the 15-kD Escherichia coli DNA binding protein H-NS. *Molecular Microbiology*, 2(3):323–329, 1988.

[36] Roy M Williams and Sylvie Rimsky. Molecular aspects of the E. coli nucleoid protein, H-NS: a central controller of gene regulatory networks. *FEMS Microbiology Letters*, 156(2):175–185, 1997.

[37] Remus Thei Dame, Claire Wyman, and Nora Goosen. H-NS mediated compaction of DNA visualised by atomic force microscopy. *Nucleic Acids Research*, 28(18):3504–3510, 2000.

[38] Charles J Dorman. H-NS: a universal regulator for a dynamic genome. *Nature Reviews Microbiology*, 2(5):391–400, 2004.

[39] Yingjie Liu, Hu Chen, Linda J Kenney, and Jie Yan. A divalent switch drives H-NS/DNA-binding conformations between stiffening and bridging modes. *Genes & Development*, 24(4):339–344, 2010.

[40] Ramon A van der Valk, Jocelyne Vreede, Liang Qin, Geri F Moolenaar, Andreas Hofmann, Nora Goosen, and Remus T Dame. Mechanism of environmentally driven conformational changes that modulate H-NS DNA-bridging activity. *Elife*, 6:e27369, 2017.

[41] Heisaburo Shindo, Takanobu Iwaki, Ryoichi Ieda, Hitoshi Kurumizaka, Chiharu Ueguchi, Takeshi Mizuno, Soichi Morikawa, Haruki Nakamura, and Hitoshi Kuboniwa. Solution structure of the DNA binding domain of a nucleoid-associated protein, H-NS, from Escherichia coli. *FEBS Letters*, 360(2):125–131, 1995.

[42] Charles J Dorman, Jay CD Hinton, and Andrew Free. Domain organization and oligomerization among H-NS-like nucleoid-associated proteins in bacteria. *Trends in Microbiology*, 7(3):124–128, 1999.

[43] Ramon A Van Der Valk, Jocelyne Vreede, Frédéric Crémazy, and Remus T Dame. Genomic looping: a key principle of chromatin organization. *Journal of Molecular Microbiology and Biotechnology*, 24(5-6):344–359, 2015.

[44] James A Maier, Carmenza Martinez, Koushik Kasavajhala, Lauren Wickstrom, Kevin E Hauser, and Carlos Simmerling. ff14SB: improving the accuracy of protein side chain and backbone parameters from ff99SB. *Journal of Chemical Theory and Computation*, 11(8):3696–3713, 2015.

[45] Ivan Ivani, Pablo D Dans, Agnes Noy, Alberto Pérez, Ignacio Faustino, Adam Hospital, Jürgen Walther, Pau Andrio, Ramon Goñi, Alexandra Balaceanu, et al. Parmbsc1: a refined force field for DNA simulations. *Nature Methods*, 13(1):55–58, 2016.

[46] William L. Jorgensen, Jayaraman Chandrasekhar, Jeffry D. Madura, Roger W. Impey, and Michael L. Klein. Comparison of simple potential functions for simulating liquid water. *The Journal of Chemical Physics*, 79(2):926–935, 1983.

[47] TE III Cheatham, JL Miller, T Fox, TA Darden, and PA Kollman. Molecular dynamics simulations on solvated biomolecular systems: the particle mesh Ewald method leads to stable trajectories of DNA, RNA, and proteins. *Journal of the American Chemical Society*, 117(14):4193–4194, 1995.

[48] Ulrich Essmann, Lalith Perera, Max L Berkowitz, Tom Darden, Hsing Lee, and Lee G Pedersen. A smooth particle mesh Ewald method. *The Journal of Chemical Physics*, 103(19):8577–8593, 1995.

[49] David Van Der Spoel, Erik Lindahl, Berk Hess, Gerrit Groenhof, Alan E Mark, and Herman JC Berendsen. GROMACS: fast, flexible, and free. *Journal of Computational Chemistry*, 26(16):1701–1718, 2005.

[50] Mark James Abraham, Teemu Murtola, Roland Schulz, Szilárd Páll, Jeremy C Smith, Berk Hess, and Erik Lindahl. GROMACS: High performance molecular simulations through multi-level parallelism from laptops to supercomputers. *SoftwareX*, 1:19–25, 2015.

[51] Berk Hess, Henk Bekker, Herman JC Berendsen, and Johannes GEM Fraaije. LINCS: a linear constraint solver for molecular simulations. *Journal of Computational Chemistry*, 18(12):1463–1472, 1997.

[52] Shuichi Miyamoto and Peter A Kollman. Settle: An analytical version of the SHAKE and RATTLE algorithm for rigid water models. *Journal of Computational Chemistry*, 13(8):952–962, 1992.

[53] Giovanni Bussi, Davide Donadio, and Michele Parrinello. Canonical sampling through velocity rescaling. *The Journal of Chemical Physics*, 126(1):014101, 2007.

[54] Michele Parrinello and Aneesur Rahman. Polymorphic transitions in single crystals: A new molecular dynamics method. *Journal of Applied Physics*, 52(12):7182–7190, 1981.

[55] Shuichi Nosé and ML Klein. Constant pressure molecular dynamics for molecular systems. *Molecular Physics*, 50(5):1055–1076, 1983.
